# Drought history affects grassland plant and microbial carbon turnover during and after a subsequent drought event

**DOI:** 10.1111/1365-2745.12593

**Published:** 2016-05-24

**Authors:** Lucia Fuchslueger, Michael Bahn, Roland Hasibeder, Sandra Kienzl, Karina Fritz, Michael Schmitt, Margarete Watzka, Andreas Richter

**Affiliations:** ^1^ Department of Microbiology and Ecosystem ScienceUniversity of ViennaAlthanstrasse 14A‐1090ViennaAustria; ^2^ Institute of EcologyUniversity of InnsbruckSternwartestrasse 15A‐6020InnsbruckAustria; ^3^ Present address: National Institute for Amazonian Research (INPA)Av. André Araujo 2936AleixoManausAmazonasCEP: 69067‐375Brazil

**Keywords:** ^13^C pulse labelling, below‐ground carbon allocation, drought, microbial community composition, nitrogen, phospholipid fatty acid, plant–soil (below‐ground) interactions, recovery, resilience

## Abstract

1. Drought periods are projected to become more severe and more frequent in many European regions. While effects of single strong droughts on plant and microbial carbon (C) dynamics have been studied in some detail, impacts of recurrent drought events are still little understood.
2. We tested whether the legacy of extreme experimental drought affects responses of plant and microbial C and nitrogen (N) turnover to further drought and rewetting. In a mountain grassland, we conducted a ^13^C pulse‐chase experiment during a naturally occurring drought and rewetting event in plots previously exposed to experimental droughts and in ambient controls (AC). After labelling, we traced ^13^C below‐ground allocation and incorporation into soil microbes using phospholipid fatty acid biomarkers.
3. Drought history (DH) had no effects on the standing shoot and fine root plant biomass. However, plants with experimental DH displayed decreased shoot N concentrations and increased fine root N concentrations relative to those in AC. During the natural drought, plants with DH assimilated and allocated less ^13^C below‐ground; moreover, fine root respiration was reduced and not fuelled by fresh C compared to plants in AC.
4. Regardless of DH, microbial biomass remained stable during natural drought and rewetting. Although microbial communities initially differed in their composition between soils with and without DH, they responded to the natural drought and rewetting in a similar way: gram‐positive bacteria increased, while fungal and gram‐negative bacteria remained stable. In soils with DH, a strongly reduced uptake of recent plant‐derived ^13^C in microbial biomarkers was observed during the natural drought, pointing to a smaller fraction of active microbes or to a microbial community that is less dependent on plant C.
5. *Synthesis*. Drought history can induce changes in above‐ vs. below‐ground plant N concentrations and affect the response of plant C turnover to further droughts and rewetting by decreasing plant C uptake and below‐ground allocation. DH does not affect the responses of the microbial community to further droughts and rewetting, but alters microbial functioning, particularly the turnover of recent plant‐derived carbon, during and after further drought periods.

## Introduction

Climate projections for Central Europe predict severe alterations in rainfall timing at largely constant annual precipitation; thus, the frequency of extreme weather events, such as droughts and torrential rainfall events, is very likely going to increase (Field *et al*. 2012; IPCC 2013; Gobiet *et al*. 2014). Such extreme climatic fluctuations have been suggested to be more challenging than gradual changes in mean climate for plants and possibly also for microbes (Schimel, Balser & Wallenstein 2007; Smith 2011; Reyer *et al*. 2013; Hoover, Knapp & Smith 2014).

Plants have developed complex morphological, physiological and biochemical adaptions to reduce and adjust to water stress and its consequences for plant fitness (Iljin 1957; Chaves *et al*. 2002; Chaves, Maroco & Pereira 2003; Muller *et al*. 2011; Manzoni *et al*. 2013; McDowell *et al*. 2013; Reyer *et al*. 2013; Osakabe *et al*. 2014). Extreme droughts have been found lead to reductions in plant growth and CO_2_ uptake, thereby decreasing the carbon (C) sink potential of ecosystems (Ciais *et al*. 2005; Reichstein *et al*. 2013; Hoover, Knapp & Smith 2014; Frank *et al*. 2015). Moreover, drought periods can severely alter the quantity and quality of carbon inputs to the soil: they can alter C:N ratios of leaf and root litter (Walter *et al*. 2012; Sanaullah *et al*. 2014; García‐Palacios *et al*. 2015) and reduce below‐ground C allocation (Ruehr *et al*. 2009; Sanaullah, Chabbi & Rumpel 2012; Fuchslueger *et al*. 2014a; Canarini & Dijkstra 2015; Hasibeder *et al*. 2015), which can in turn affect microbe‐mediated C turnover (Bardgett *et al*. 2013; Canarini & Dijkstra 2015; García‐Palacios *et al*. 2015).

Soil micro‐organisms operate on much smaller spatial and faster temporal time‐scales than plants (Wolters *et al*. 2000; Prosser *et al*. 2007). During droughts, microbial activity has been shown to decrease (Jensen *et al*. 2003; Alster *et al*. 2013), as microbes have to avoid desiccation and mortality by adapting to increasing osmotic conditions, and to decreased substrate availability caused by reduced diffusion (Or *et al*. 2007; Schimel, Balser & Wallenstein 2007). They can switch to dormant or permanent states (e.g. producing cysts, see Lennon & Jones 2011), or, like mycorrhizas, can trade water from soil micropores for labile plant carbon (Allen 2007). As a result, drought periods have been shown to induce shifts within the active microbial community promoting drought‐tolerant generalists, for example some gram‐positive bacterial or fungal groups (Yuste *et al*. 2011; Lennon *et al*. 2012; de Vries *et al*. 2012a; Fuchslueger *et al*. 2014a).

However, impacts of drought not only depend on the tolerance or resistance of organisms to drought events, but also depend on their resilience, that is the ability to recover (Orwin & Wardle 2004; Allison & Martiny 2008; Smith 2011; Shade *et al*. 2012; Griffiths & Philippot 2013; de Vries & Shade 2013; Hodgson, McDonald & Hosken 2015). Plants are often able to recover upon rewetting (e.g. see Xu, Zhou & Shimizu 2010), but drought effects can also be carried over and reduce plant growth or increase mortality in the following growing seasons (Anderegg *et al*. 2015) and eventually might lead to changes in plant community composition (Kardol *et al*. 2010; Van der Molen *et al*. 2011; Hoover, Knapp & Smith 2014; Rammig *et al*. 2015). Alternatively, recurring extreme droughts can also increase the resistance (e.g. of grass species) to subsequent drought events (Walter *et al*. 2011).

Also microbes have been shown to become potentially active after drought within hours upon rewetting (Barnard, Osborne & Firestone 2015); they can resuscitate from dormancy (Schimel, Balser & Wallenstein 2007; Lennon & Jones 2011), and depending on the duration of drought, they start to regrow within one to several days (Meisner, Bååth & Rousk 2013; Blazewicz, Schwartz & Firestone 2014; Meisner, Rousk & Bååth 2015). Therefore, rewetting water pulses and accumulated substrate as well as microbial growth fuel mostly short‐lived activity pulses in C and nitrogen (N) mineralization (Birch 1958; Denef *et al*. 2001; Fierer & Schimel 2003; Schimel, Balser & Wallenstein 2007; Borken & Matzner 2009; Placella, Brodie & Firestone 2012). Eventually, recurring drying and rewetting of soils have been shown to foster a more drought‐tolerant microbial baseline community (Fierer, Schimel & Holden 2003; de Vries *et al*. 2012b; Evans & Wallenstein 2014), which could be accompanied by shifts in microbial functioning (reviewed by Krause *et al*. 2014; but see Evans & Wallenstein 2012).

While effects of single droughts and subsequent rewetting have been comparatively well studied, there are still major gaps in our understanding of the legacies of droughts on the responses of plants, microbes and their interactions to subsequent droughts. The projected increase in the frequency of drought and rewetting events could both decrease or enhance the sensitivity of organisms to similar disturbances (Reichstein *et al*. 2013; Walter *et al*. 2013; Hawkes & Keitt 2015). It will likely cause different responses of above‐ and below‐ground organisms (Bardgett *et al*. 2013) and has been suggested to reduce the stabilization of C from rhizodeposition in the microbial biomass and thus in soils (Canarini & Dijkstra 2015). In this context, evidence from intact plant–soil systems is scarce, and it is still not clear whether and how a history of droughts alters responses of plant and soil microbial C turnover to a subsequent drought under *in situ* conditions. To address this gap, our study investigated whether the legacy of previous droughts changed the responses of plants, soil microbes and the turnover of recent plant‐derived C to a subsequent drought and rewetting in a mountain grassland. We conducted a ^13^C pulse‐chase experiment during a naturally occurring drought and rewetting event in plots that had been previously exposed to experimental droughts and in plots without drought history (DH) (ambient controls, AC). This permitted tracing the transfer of assimilated ^13^C from plants below‐ground, to the extractable soil organic carbon pool and further into microbial phospholipid fatty acid (PLFA) biomarkers and thus into growing micro‐organisms. We hypothesized that DH would lead to (i) an overall increase in root‐to‐shoot ratios, increased C:N ratios in both roots and shoot, and (ii) a decreased uptake and allocation of C below‐ground during a subsequent drought. In view of the high resilience of the microbial community composition in the studied grassland to severe drought (Fuchslueger *et al*. 2014a,b), we expected (iii) that there would be no legacy effects of DH on microbial community composition. However, we expected that (iv) DH would affect microbial carbon uptake and turnover of plant‐derived C to a subsequent drought and rewetting.

## Materials and methods

### Site Description

The study area is located in the Austrian Central Alps near Neustift, Stubai Valley (47°07′45″N, 11°18′20″E). The sampling site is situated at 1850 m and part of a mountain meadow that is cut annually for hay production at peak biomass (late July/early August), is fertilized every 3–4 years and is grazed for a few days in spring and autumn. The soil is a dystric cambisol (FAO‐soil classification system) with a pH (in CaCl_2_) of 5.4, the vegetation is dominated by highly productive perennial grasses and herbs, and the meadow is generally characterized by comparatively high primary productivity and high soil CO_2_ efflux rates (Bahn *et al*. 2006, 2010; Schmitt *et al*. 2010). The mean annual temperature and the mean annual precipitation are 3 °C and 1100 mm, respectively.

### Experimental Set‐Up – ‘Drought History’ Treatment

**Figure 1:**
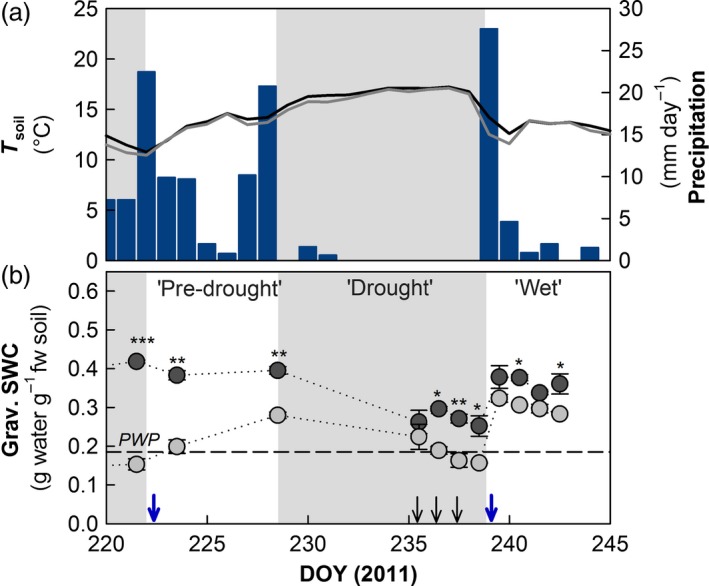
Microclimatic conditions during the experimental period in 2011. (a) Daily means of soil temperature (*T*_soil_) at 10 cm soil depth in ambient controls (AC, black line) and plots with drought history (DH, grey line) and daily sums of precipitation in mm (blue bars). (b) Gravimetrically determined soil water content (SWC, g water g^−1^ fresh soil; *n* = 3; ± SE) AC plots (black circles) and in DH plots (grey circles); differences at single samplings were assessed by paired *t*‐test after Bonferroni correction (asterisks mark levels of significance: *P < 0.05, **P < 0.01, ***P < 0.001). The dashed line marks the SWC equalling to the permanent wilting point (PWP) for plants at a pF of 4.2. Blue arrows indicate the end of the experimental and the natural drought, black arrows indicate the three subsequent days of ^13^C pulse labelling. The grey background marks periods of experimental rain exclusion and the natural drought.

The experiment was conducted on plots that had been repeatedly exposed to extreme drought periods, which in the following will be referred to as the ‘DH’ treatment, as well as on control plots receiving naturally occurring amounts of precipitation, referred to as ‘AC’. In DH plots, rainout shelters (3 × 2 m, *n* = 3) had been installed for 8–10 weeks during the growing seasons of three consecutive years (2009, 2010 and 2011, respectively), excluding approximately one‐third of the annual precipitation in each of the years. The period of precipitation removal targeted a significant reduction in soil moisture for a period of 1 month, corresponding to a drought event with a return interval of 1000 years, as based on available climate records. Drought was terminated in defined and coordinated manner by simulating a heavy rainfall event, applying 20 mm of previously collected rain water to each of the plots when the rainout shelters were removed and then exposed to natural precipitation. This caused soil moisture to rise again above the permanent wilting point (Fig. 1).

In 2011, DH plots were equipped with rainout shelters from May 31 until rewetting on August 9. After rewetting, during the recovery period following the experimental drought, the soil water content (SWC) in DH plots doubled from 0.15 up to 0.29 g water g^−1^ fresh soil, whereas it remained in the range of 0.4 g g^−1^ in AC plots (‘pre‐drought’, Fig. 1). This period was followed by a natural drought, with < 1‐mm precipitation falling during a period of 10 consecutive days, causing soil moisture to decrease sharply in both DH and AC plots (‘drought’, Fig. 1). The natural drought was terminated by a short, intensive rainy period, leading to SWC in the range of 0.3–0.4 g g^−1^ in both DH and AC plots (‘wet’, Fig. 1).

### Pulse Labelling Procedure

Towards the end of the natural drought, in each of the three AC and the three DH plots, an area of 1 × 1 m was pulse‐labelled with 99.9 at% ^13^CO_2_ over a period of 90 min. Pulse labelling was carried out during the late morning hours of three consecutive days with bright weather conditions and similarly high photosynthetic radiation (23–25 August 2011, see Table S1 in Supporting information). A detailed description of the labelling set‐up and procedure is given in Bahn *et al*. (2013) and in Fuchslueger *et al*. (2014a).

### Sampling of Plant and Soil Material

Sampling of plant biomass (shoots and fine roots) and soil was performed 1 h before labelling to determine the respective natural abundances of ^13^C to calculate ^13^C excess and then seven times after pulse labelling (1.5, 3.5, 6.5, 25.5, 48, 96 and 144 h after ^13^C pulse). The rainfall event at the end of the natural drought occurred between 48 and 96 h after pulse labelling (Fig. 1); therefore, the last two samplings were carried out during the ‘wet’ period. In addition, a set of soil samples were taken before the natural drought (‘pre‐drought’), permitting to identify the effects of experimental DH.

For each plant and soil sample, materials from two collars with an area of 5 × 7 cm and 10 cm soil depth, carefully cut out using a knife, were pooled. From each collar, above‐ground plant material (shoots) was cut and collected, the uppermost organic material was removed and the soil was carefully sieved to 2 mm and manually freed from fine roots. Aliquots of soil samples were immediately frozen at −80 °C for the determination of PLFAs, and the remaining soil was stored at 4 °C until further processing. Fine roots were collected, carefully washed and coarsely dried with paper towels. Aliquots of fine roots were further used to determine fine root respiration (for a detailed description see Hasibeder *et al*. 2015). Shoots and remaining fine roots were treated by microwave for 3 min to interrupt metabolic activity (Popp *et al*. 1996). Plant materials and soil aliquots were dried at 60 °C for 72 h, weighed and finely ground for subsequent analyses of bulk ^13^C, ^15^N and total C and N contents by EA‐IRMS (EA 1110 (CE Instruments, Milan, Italy), coupled to a Finnigan MAT Delta Plus IRMS (Thermo Fisher Scientific, Waltham, MA, USA).

### Determination of Extractable Organic Carbon and Soil Microbial Biomass

Extractable organic carbon (EOC) and total extractable nitrogen (N) fractions were determined in K_2_SO_4_ extracts (2 g of fresh soil was extracted with 20 mL of 0.5 m K_2_SO_4_) by a TOC/TN analyser (TOC‐V CPH E200V/TNM‐122V; Shimadzu, Vienna, Austria). The δ^13^C of the EOC pool was measured by direct injection (without column, direct mode) and at a flow of 0.5 mL water min^−1^ on a high‐performance liquid chromatography (HPLC) (Dionex Corporation, Sunnyvale, CA, USA) linked to a Finnigan Delta V Advantage Mass Spectrometer, connected by a Finnigan LC‐IsoLink Interface (both Thermo Fisher Scientific). ${\text{NH}}_{4}^{+}$ concentrations were measured photometrically from K_2_SO_4_ extracts using a modified indophenol reaction method (Kandeler & Gerber 1988). ${\text{NO}}_{3}^{-}$ was measured from water extracts (2 g of fresh soil was extracted with 20 mL of MilliQ water) by chemically suppressed ion chromatography (DX500, Dionex, Vienna, Austria) on a Dionex AS11 column. Extractable organic nitrogen (EON) was calculated by subtracting inorganic N pools (${\text{NH}}_{4}^{+}$ and ${\text{NO}}_{3}^{-}$) from total extractable N.

Soil microbial biomass and microbial community composition, as well as microbial incorporation of plant‐derived carbon, were estimated by extracting PLFAs from frozen soil samples using the same procedure as described in Fuchslueger *et al*. (2014a). Total lipids were extracted from soil using a chloroform/methanol/citric acid buffer and cleaned from neutral lipids. After an internal standard (19:0) was added, PLFAs were converted to fatty acid methyl esters (FAMEs) by alkaline methanolysis. Samples were analysed using a Trace GC Ultra connected by a GC‐IsoLink to a Delta V Advantage Mass Spectrometer (all Thermo Fisher Scientific). Samples were injected in splitless mode (injector temperature 220 °C) and separated on a DB23 column (60 m × 0.25 mm × 0.25 μm; Agilent, Vienna, Austria) with 1.5 mL min^−1^ He as the carrier gas (GC programme: 1.5 min at 70 °C, 30 °C m^−1^ at 150 °C, 1 min at 150 °C, 4 °C min^−1^ at 230 °C and 15 min at 230 °C). FAMEs were identified using mixtures of bacterial and fungal FAMEs (bacterial acid methyl ester mix and 37 Comp. FAME Mix; Supelco, Bellefonte, PA, USA). FAMEs were quantified against the internal standard (19:0) and corrected for the methyl group that was added during methylation. We used the markers 18:1ω9, 18:2ω6,9, 18:3ω3,6,9 and 16:1ω5 for total fungi. However, 16:1ω5 is a marker used either for arbuscular mycorrhizal fungi or for gram‐negative bacteria (Zelles 1997; Olsson 2006). The sum of i15:0, a15:0, i16:0 a16:0 and a17:0 was used as gram‐positive bacterial marker and 16:1ω7, 18:1ω7, cy17:0(9/10) and cy19:0(9/10) as gram‐negative bacteria. Gram‐positive, gram‐negative and 15:0, 17:1ω6, 17:9, 18.1ω5 and 10Me18:0 markers were summed to give total bacterial PLFAs.

### Calculation of ^13^C Excess and Turnover

The excess ^13^C, that is the pulse labelling‐derived amount of ^13^C (given in μmol or nmol ^13^C g^−1^ dm plant biomass or soil), was calculated for the available carbon pools as follows:

(eqn 1) $$\text{excess}{\;}^{13}C=\left(\text{atom}{\%}_{\mathrm{sample}}-\text{atom}{\%}_{\mathrm{nat}.\mathrm{ab}}\right)*\frac{C_{\mathrm{pool}}}{100}$$

with atom%_sample_ describing the atom% ^13^C of the labelled sample, atom%_nat.ab_ representing the natural ^13^C abundance of these samples (taken before labelling) and *C*_pool_ being the C concentrations of the respective sampled pool (i.e. shoots, fine roots, fine root respiration, EOC and PLFAs). The C turnover in plant, soil and microbial pools underlies different temporal dynamics. We therefore used different models to calculate the mean residence time of ^13^C in respective compartments. A detailed description of the different models is given in the Supporting information (Appedix S1).

### Statistical Analyses

Effects of DH and the responses to the natural drought and subsequent rewetting on plant biomass, as well as on plant, soil and microbial carbon and/or nitrogen pools were assessed by two‐way analysis of variance (anova) followed by a Tukey's HSD *post hoc* test. If data did not meet anova assumptions, they were either log‐transformed or rank‐normalized. To display the overall effects of DH, as well as of the natural drought and subsequent rewetting on microbial community dynamics in relation to changes in soil parameters, a canonical correspondence analysis (CCA) was conducted. The relative abundances of single PLFA markers were used as community ‘matrix’, and as environmental ‘matrix’, we used a range of soil parameters (including SWC, *T*_soil_ at 10 cm, EOC, EON, ${\text{NH}}_{4}^{+}$ and ${\text{NO}}_{3}^{-}$ concentrations). CCA scores were analysed for the effects of experimental DH and its consequences for soil responses to the natural drought and subsequent rewetting by two‐way permutational anova. Effects of DH on the ^13^C‐turnover dynamics after pulse labelling were analysed by two‐way repeated‐measures anova. In addition, paired *t*‐tests were calculated for single time points after the pulse labelling and Bonferroni corrected). All statistical analyses were performed in r.3.0.3 (R Development Core Team, 2013) using the vegan package to compute the CCA (Oksanen *et al*. 2013) and in sigmaplot 11 (Systat Software, San Jose, CA, USA).

## Results

### Microclimatic Parameters

After the end of the experimental drought completing the DH pre‐treatment, the meadow received in total 58 mm of precipitation increasing the SWC from 0.15 to 0.29 g water g^−1^ fresh soil in the DH plots. However, the SWC was still significantly lower than in the AC, which had not been pre‐exposed to severe experimental drought (Fig. 1). The subsequent natural drought decreased the SWC in DH plots from 0.29 to 0.16 g water g^−1^ fresh soil and thus to values below the permanent wilting point (at 0.18 g water g^−1^ fresh soil); in AC plots, SWC was reduced from 0.40 to 0.25 g water g^−1^ fresh soil. After 10 consecutive days without rainfall, a heavy precipitation event of 22 mm strongly increased the SWC in both DH and AC plots to values above 0.3 g water g^−1^ fresh soil. Because of these strong microclimatic dynamics samples were categorized to ‘pre‐drought’ (after the rain exclusion), ‘drought’ (during the natural drought) and ‘wet’ (after the subsequent rewetting by the rain pulse; Fig. 1, Table 1). One drawback of our study is that in DH plots, soil moisture had not recovered to the same levels as in the AC plots when the natural drought started. Thus, we cannot distinguish legacy effects of DH on biota and their drought responses from effects that related to the intensity of drought.

**Table 1:** Effects of drought history (DH) and natural drought and rewetting (‘pre‐drought’, ‘drought’, ‘wet’) were determined by two‐way anova. Plant samples were collected during the natural drought period (‘drought’) and after rewetting (‘wet’; in total 8 samplings). Soil sampling started already before the natural drought (including ‘pre‐drought’ samples)

	DH		Drought‐rewetting		Interaction	
	*F*_1_	*P*	*F*_1_	*P*	*F*_1,1_	*P*
Plant parameters						
Shoot BM (g dw m^−2^)	1.0	ns	0.8	ns	0.1	ns
Fine root BM (g dw m^−2^)	0.1	ns	5.9	*	0.1	ns
Shoot C (mmol C g^−1^ dw)	1.9	ns	7.4	**	0.1	ns
Shoot N (mmol N g^−1^ dw)	19.1	***	0.5	ns	1.9	ns
Shoot C:N ratio	60.4	**	4.7	*	1.9	ns
Fine root C (mmol C g^−1^ dw)	2.4	ns	0.4	ns	0.9	ns
Fine root N (mmol N g^−1^ dw)	15.5	***	0.1	ns	1.1	ns
Fine root C:N ratio	10.7	***	0.4	ns	1.4	ns
Fine root resp. (μmol C g^−1^ dw h^−1^)	23.6	***	2.8	ns	1.3	ns
	*F*_1_	*P*	*F*_2_	*P*	*F*_1,2_	*P*
Soil parameters						
SWC (%)	79.4	***	92.1	***	10.0	***
*T*_soil_ (°C)	23.6	***	59.9	***	0.9	ns
Soil C_tot_ (%)	0.1	ns	0.5	ns	0.1	ns
Soil N_tot_ (%)	0.3	ns	0.5	ns	0.1	ns
EOC (μmol C g^−1^ dw soil)	9.6	**	1.8	ns	1.5	ns
EON (μmol N g^−1^ dw soil)	2.6	ns	3.9	*	2.1	ns
${\text{NH}}_{4}^{+}$ (μmol N g^−1^ dw soil)	0.1	ns	15.4	***	0.2	ns
${\text{NO}}_{3}^{-}$ (μmol N g^−1^ dw soil)	7.6	**	0.2	ns	1.0	ns
Total PLFAs (μmol C g^−1^ dw soil)	0.9	ns	2.5	ns	0.7	ns
Fungi (μmol C g^−1^ dw soil)	1.4	ns	1.4	ns	0.8	ns
Total bacteria (μmol C g^−1^ dw soil)	0.9	ns	3.7	*	1.3	ns
Gram positive (μmol C g^−1^ dw soil)	0.7	ns	7.9	**	1.5	ns
Gram negative (μmol C g^−1^ dw soil)	1.0	ns	2.5	ns	1.1	ns
Fungi:bacteria ratio	0.1	ns	16.9	***	10.1	***

BM, biomass; SWC, gravimetric soil water content in the upper 10 cm of soil; *T*_soil_, soil temperature at 10 cm soil depth; EOC, extractable organic C; EON, extractable organic N; total PLFAs, total phospholipid fatty acids; fungi: total fungal PLFA markers; total bacteria: total bacterial PLFA markers; Gram positive and Gram negative: gram‐positive and gram‐negative bacterial PLFA markers; fungi:bacteria: the ratio of total fungal to bacterial PLFA markers.

Asterisks mark levels of significance (*n* = 3, ns, not significant, *P < 0.05; **P < 0.01; ***P < 0.001).

### Effects of Drought History on Plant Responses to a Natural Drought and Rewetting Event

**Figure 2:**
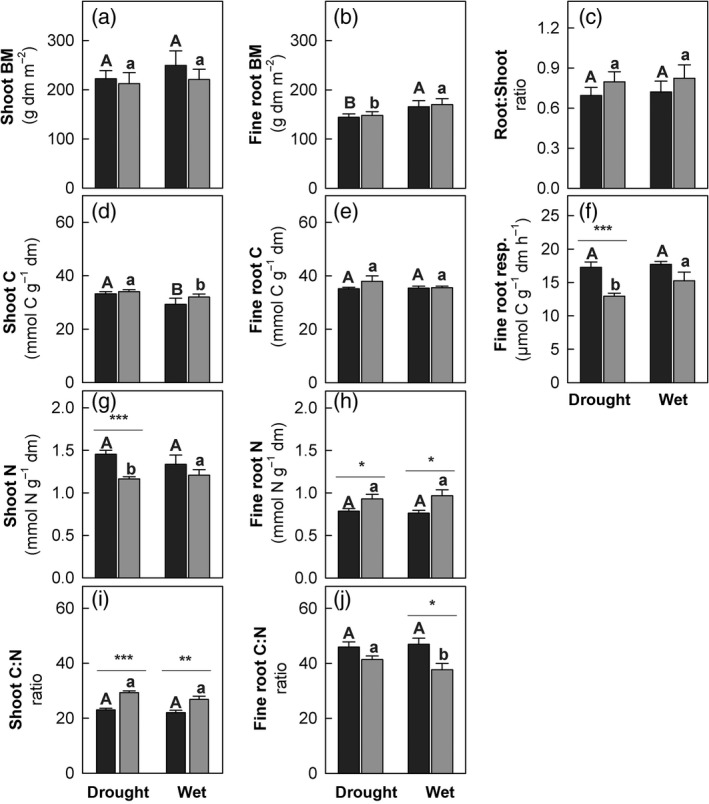
Plant parameters in ambient controls (AC, black bars) and drought history (DC, grey bars) plots and during the natural drought (‘drought’) and rewetting (‘wet’) event. (a) Shoot biomass, (b) fine root biomass and (c) root:shoot ratios; (d) shoot and (e) fine root carbon concentrations, respectively, (f) fine root respiration rates; (g) shoot and (h) fine root nitrogen concentrations, respectively, and (i) shoot and (j) fine root C:N ratios, respectively (*n* = 3, error bars = SE). Significant differences between DH and AC treatment are marked with asterisks (*P < 0.05; **P < 0.01; ***P < 0.001); differences between dry and wet conditions within DH treatment are marked with lower case letters and within AC with upper case letters (at a 0.05 significance level) (see Table 1 for more details).

Plant shoot biomass was not affected by DH and showed no significant response to the subsequent natural drought and rewetting (Fig. 2, Table 1). Also fine root biomass was similar in all plots during the natural drought, but significantly increased in both DH and AC plots after rewetting (Fig. 2, Table 1). Changes in the root:shoot biomass ratios were, however, not significant (Fig. 2, Table 1).

Independent of DH, C concentrations in plant shoots decreased significantly during the ‘wet’ period following the natural drought, whereas fine root C concentrations remained unchanged. The N concentrations in turn were significantly lower in shoots, but higher in fine roots of plants in DH compared to AC plots throughout the study period. This resulted in higher shoot, but lower fine root C:N ratios in plants in DH than in AC plots (Fig. 2, Table 1). DH led to a decrease of fine root respiration during the natural drought by 25% compared to AC, but this effect was no longer significant after the subsequent rewetting (Fig. 2, Table 1).

**Figure 3:**
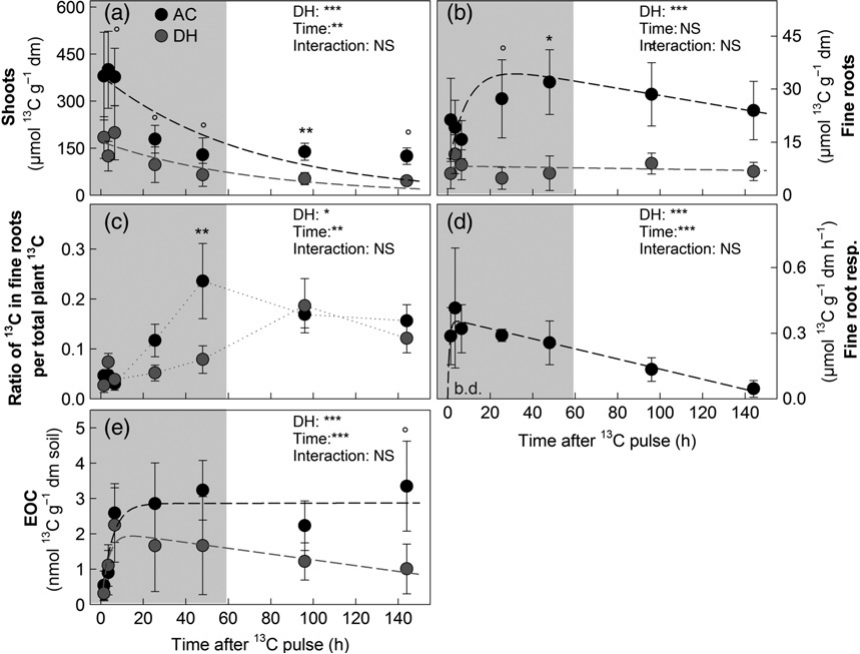
Pulse labelling‐derived ^13^C (i.e. excess ^13^C) in ambient controls (AC, black circles) and drought history (DH, grey circles) plots over time in (a) plant shoots, (b) fine root biomass, (c) the proportion of ^13^C excess in fine roots per total plant ^13^C excess (sum of shoot and fine roots), ^13^C excess in (d) fine root respiration (b.d. below detection limit), (e) soil extractable organic C pool (means; *n* = 3, ± SE, respectively). In (a–e) nonlinear regressions (dashed lines) describe the ^13^C dynamics in AC (black) and DH plots (grey). The grey background indicates samples taken during natural drought; the other samples were taken after subsequent rewetting. Effects of DH treatment and time after labelling were assessed by two‐way repeated‐measures anova; asterisks mark significant differences at single samplings (paired *t*‐tests, Bonferroni corrected; °*P* < 0.1; *P < 0.05, **P < 0.01; ***P < 0.001) (See Table 2 and Table S2 for detailed statistical information and model fittings, respectively).

The ^13^C pulse‐chase labelling experiment was conducted during the natural drought period, and sampling continued also after rewetting. The amount of ^13^C label assimilated by shoots was significantly lower in DH than in AC plots. Nonetheless, in all plots, ^13^C peaked within the first 6.5 h after the labelling pulse, followed by an exponential decrease over time with mean residence times (MRT) of ^13^C of about 69 h (Fig. 3, Table 2). In fine roots from DH plots, ^13^C peaked earlier (after 3.5 h), but this peak was significantly lower, and the MRT of ^13^C was 3.5 times longer as compared to AC plots (Fig. 3, Table 2). In AC plots, the proportion of ^13^C in fine roots to ^13^C in total plant material (i.e. the sum of excess ^13^C in shoots and fine roots) was highest 48 h after the labelling pulse, but overall, this proportion was significantly decreased in DH plots (Fig. 3, Table 2). Moreover, in DH plots, no ^13^C was detectable in fine root respiration, while in AC plots, there was a clear ^13^C signal, which peaked already after 3.5 h after labelling and then followed a linear decrease (Fig. 3, Table 2).

**Table 2:** Effects of drought history (DH) on ^13^C dynamics in plant, soil and soil microbial carbon pools during the natural drought and rewetting event. Differences between DH plots and ambient controls (AC) were assessed by two‐way repeated‐measures anova, using DH and time after labelling as factors, and plot numbers as within factor (*n* = 3)

	Two‐way repeated‐measures anova						AC ^13^C dynamics					DH ^13^C dynamics				
	DH treatment		Time		Interaction		Amount at ^13^C peak	Peak time (h)	Model type	Rate constant, *k*	Mean residence times (MRT) (1 *k* ^−1^) (h)	Amount at ^13^C peak	Peak time (h)	Model type	Rate constant, *k*	MRT (1 *k* ^−1^) (h)
	*F*_1_	*P*	*F*_5_	*P*	*F*_1,5_	*P*	Mean ± SE					Mean ± SE				
Shoot BM	14.24	***	5.12	**	0.27	ns	400.3 ± 122.8	3.5	exp	0.0143	69.9	199.0 ± 86.2	6.5	exp	0.0147	69.8
Fine root BM	76.22	***	1.60	ns	2.47	ns	32.0 ± 9.2	48.0	exp‐lin	0.1017	9.8	11.6 ± 5.5	3.5	exp‐lin	0.0087	33.7
Fine root resp.	209.88	***	8.21	***	2.39	ns	0.4 ± 0.2	3.5	exp‐lin	0.0023	434.8			na		
EOC	29.83	***	12.16	***	2.37	ns	6.9 ± 3.7	48.0	exp‐lin	0.0081	123.5	2.3 ± 1.1	6.5	exp‐lin	0.0072	138.9
Total PLFAs	0.95	ns	2.50	*	4.35	**	5.4 ± 0.7	25.5	exp‐lin	0.0409	24.5	12.6 ± 4.9	144	exp	0.0240	na
Fungi	2.00	ns	3.68	**	4.17	**	1.5 ± 0.8	25.5	exp‐lin	0.0150	66.7	3.0 ± 1.2	144	exp	0.0208	na
Total bacteria	0.31	ns	2.03	ns	4.32	**	1.2 ± 0.5	48.0	exp‐lin	0.0086	116.3	4.9 ± 2.1	144	exp	0.0426	na
Gram positive	0.70	ns	1.58	ns	3.33	*	0.4 ± 0.2	3.5	exp‐lin	0.0031	332.6	1.6 ± 0.7	144	exp	0.0479	na
Gram negative	0.42	ns	2.38	ns	4.47	**	0.7 ± 0.5	25.5	exp‐lin	0.0085	117.6	2.9 ± 1.2	144	exp	0.0416	na
Fungi:bacteria	0.05	ns	1.59	ns	1.20	ns			na					na		

Significant effects are marked by asterisks (na, not available; ns, not significant; *P < 0.05; **P < 0.01; ***P<0.001). Peak time and amount of excess ^13^C are given for plant shoots and fine roots (in μmol ^13^C g^−1^ dm), fine root respiration (μmol ^13^C g^−1^ dm h^−1^), extractable organic carbon (EOC) and phospholipid fatty acids (PLFA_tot_ both in nmol ^13^C g^−1^ dm); total PLFAs were separated to fungal and bacterial, which were further divided into gram‐positive and gram‐negative bacterial markers, as well as effects on the ratio of fungal:bacterial ^13^C uptake are reported. The rate constant of ^13^C turnover (*k*) and MRT (1 *k* ^−1^; given in h) of ^13^C were determined by nonlinear regressions (*exp*: exponential decay/increase; *exp‐lin*: combination of exponential and linear regression) (see Table S2 for a more detailed description of the different models).

### Effects of Drought History on Soil Microbial and Nutrient Responses to a Natural Drought and Rewetting Event

Extractable organic carbon and nitrogen concentrations increased during the natural drought only in soils with DH. In contrast, soil inorganic N pools (${\text{NH}}_{4}^{+}$ and ${\text{NO}}_{3}^{-}$) decreased during the natural drought and slightly increased after rewetting in both DH and AC plots (Fig. S1, Table 1).

**Figure 4:**
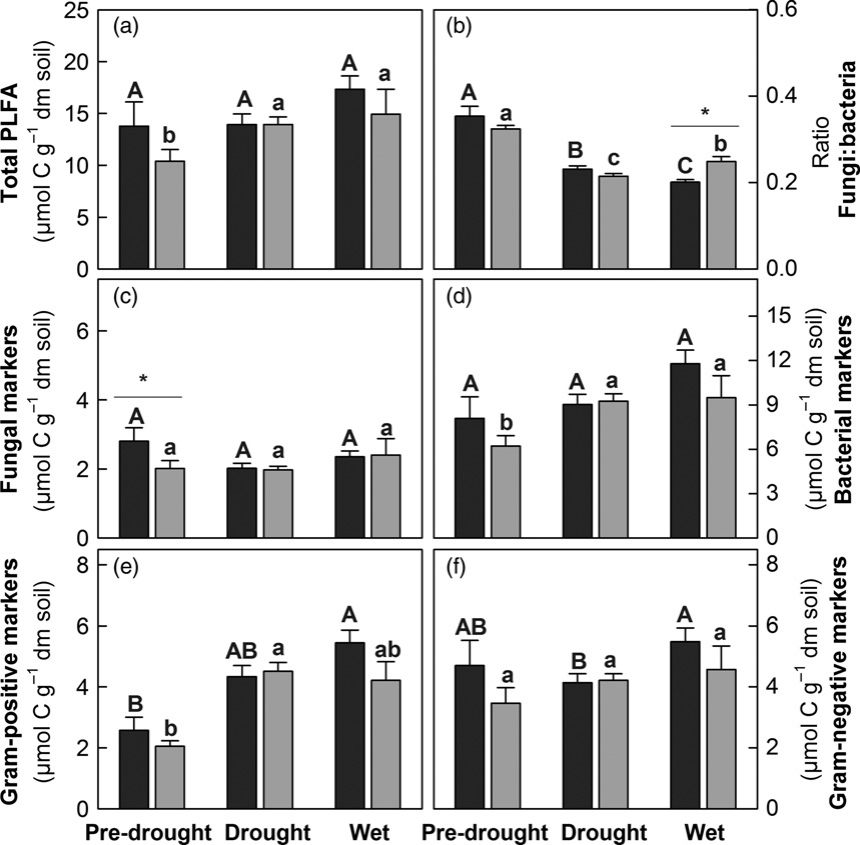
Soil microbial phospholipid fatty acid (PLFAs) markers in ambient controls (AC, black) and drought history (DH grey) plots. Samples were taken before (‘pre‐drought’), during (‘drought’) and after (‘wet’) a natural drought period. (a) Total microbial PLFAs, (b) ratio of fungal to bacterial PLFAs markers, as well as (c) fungal and (d) bacterial markers and markers for (e) gram‐positive and (f) gram‐negative bacteria are shown (*n* = 3; error bars = SE). Significant differences between DH and AC plots are marked with asterisks (*P < 0.05); differences within samples with DH treatment are marked with lower case letters, and within AC with upper case letters (at a 0.05 significance level) (see Table 1 for more details).

**Figure 5:**
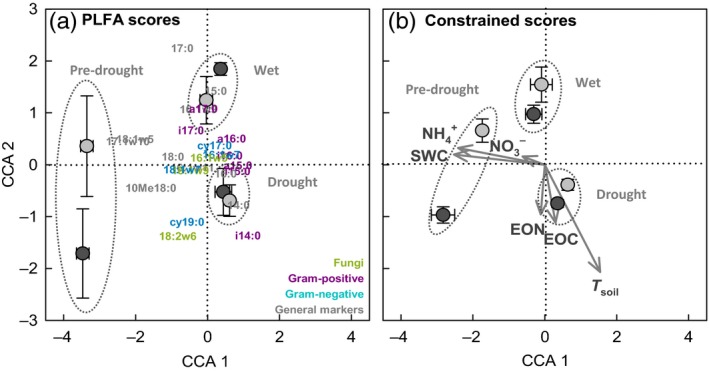
(a) Site scores of soil microbial community composition, and (b) biplot (vectors and site scores) of the constraining environmental factors determined by canonical correspondence analysis (CCA) in ambient controls (AC, black circles) and drought history (DH, grey circles) plots. As community matrix, relative abundances of phospholipid fatty acid (PLFAs) were used and combined with an environmental matrix (abbreviations are given in the text. The assignment of PLFA markers to different groups in (a) is indicated by different colours (fungi: green; gram positives: purple; gram negatives: blue; general markers: grey). Three clusters were identified (‘pre‐drought’, ‘drought’ and ‘wet’). Further statistical information is given in Table 3.

Neither in DH, nor in AC plots, the total microbial PLFA content was significantly affected by the natural drought and rewetting event (Fig. 4, Table 1). Also fungal and gram‐negative markers remained stable, while gram‐positive bacterial PLFAs increased during the natural drought period and the fungi:bacteria ratio decreased (Fig. 4, Table 1). After the subsequent rewetting, the fungi:bacteria ratio further decreased in AC plots, while in DH plots, the ratio significantly increased again (Fig. 4, Table 1). A canonical correspondence analysis confirmed that the natural drought and subsequent rewetting induced significant changes in PLFA composition in DH and AC plots. The environmental matrix (constrained variability) accounted for 41.9%, and the PLFA matrix (species scores) explained 58.1% of the total variability, of which within the species matrix, the CCA axis 1 explained 36.3% and CCA axis 2 explained 2.8%. Although DH affected constrained scores of both CCA axes 1 and 2, it had no effect on PLFA distribution. The natural drought and subsequent rewetting significantly affected both constrained and species variability forming three clusters (‘pre‐drought’, ‘drought’, ‘wet’; Fig. 5, Table 3). The species scores confirmed that fungal and gram‐negative markers were less affected by short‐term moisture variations, while gram‐positive bacterial biomarkers became more abundant during the natural drought. A biplot showed that *T*_soil_ (*R* ^2^ = 0.48, P***), SWC (*R* ^2^ = 0.44, P***), ${\text{NH}}_{4}^{+}$ (*R* ^2^ = 0.41, P***) and EON (*R* ^2^ = 0.13, P*) contributed most to the distribution of constrained scores (Fig. 5).

**Table 3:** Species (phospholipid fatty acid matrix) and constrained (environmental matrix) scores distribution for canonical correspondence analysis (CCA) axes 1 and CCA axes 2, respectively, were evaluated by two‐way permutational anova using drought history (DH) treatment and moisture levels (‘pre‐drought’, ‘drought’, ‘wet’) as factors

	Species scores				Constrained scores			
	CCA1		CCA2		CCA1		CCA2	
	*F*_1,2_	*P*	*F*_1,2_	*P*	*F*_1,2_	*P*	*F*_1,2_	*P*
DH treatment	0.3	ns	0.3	ns	6.6	*	16.4	***
Moisture level	99.4	***	13.0	**	77.4	***	68.9	***
Interaction	1.1	ns	1.9	ns	1.8	ns	3.9	*

Asterisks mark levels of significance (ns, not significant; *P < 0.05; **P < 0.01; ***P < 0.001).

### Effects of Drought History on the Microbial Turnover of Recent Plant‐Derived Carbon During a Natural Drought and Rewetting Event

**Figure 6:**
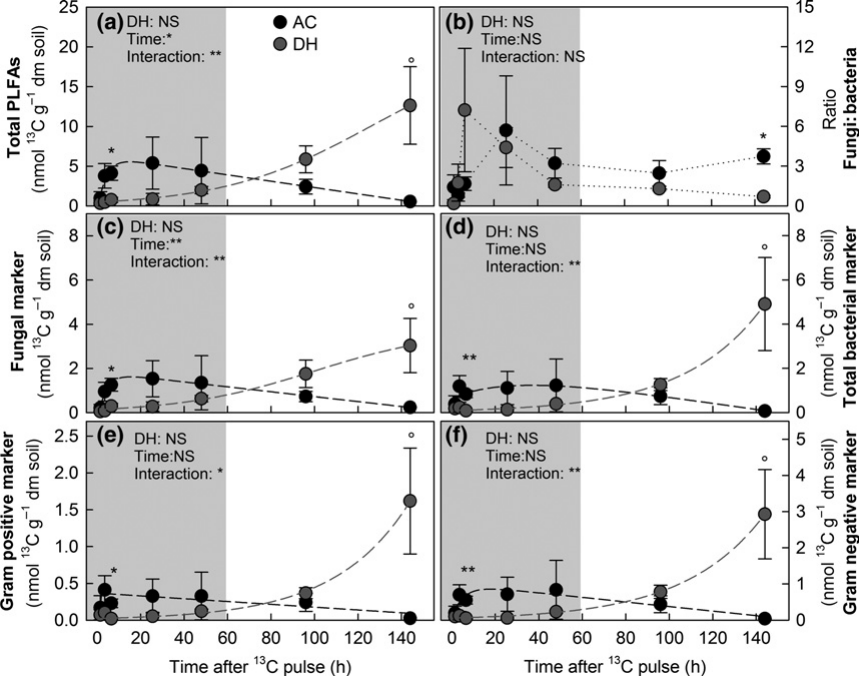
Pulse labelling‐derived ^13^C (i.e. excess ^13^C) in ambient controls (AC, black circles) and drought history (DH, grey circles) plots in (a) total phospholipid fatty acid (PLFAs) and separately for (b) total fungal and (c) total bacterial biomarker, as well as for (d) gram‐positive and (e) gram‐negative PLFAs; (means; *n* = 3*;* ± SE). Nonlinear regressions (dashed lines) describe the ^13^C dynamics in DH plots (grey) and AC (black) plots. Effects of DH on ^13^C dynamics were assessed by two‐way repeated‐measures anova; Asterisks mark significant differences at single sampling points, determined by paired *t*‐tests (Bonferroni corrected; °*P* < 0.1; *P < 0.05, **P < 0.01; ***P < 0.001) (See Table 2 and Table S2 for detailed statistical information and model fittings, respectively).

Plant‐derived ^13^C peaked 3.5 h after labelling in the EOC pool in both DH and AC plots and remained elevated for the next 45 h (Fig. 3, Table 2). The total amount of ^13^C recovered in microbial PLFAs was similar in DH and AC plots, but the turnover followed significantly different temporal dynamics (Fig. 6, Table 2). In DH plots, plant‐derived ^13^C was incorporated only slowly into soil microbial groups during drought conditions, but increased steeply in all microbial groups after rewetting, the highest values occurring at the end of the chase period (between 48 and 96 h after labelling). In contrast, in AC plots, ^13^C in PLFAs peaked much earlier (3.5–48 h after labelling), followed by a steady decreased over time. The mean residence time of ^13^C ranged from 67 h in fungi to 333 h in gram‐positive bacteria (Fig. 6, Table 2).

## Discussion

### Drought History Affects Plant C Turnover During a Natural Drought and Rewetting Event

Drought history did not induce significant changes in plant shoot and fine root biomass during a naturally occurring drought period. Similar as reported for another multi‐year drought study (Jentsch *et al*. 2011) the above‐ground biomass in our study remained stable, which is in line with a recent synthesis suggesting that multiyear precipitation reduction experiments rarely lead to a shift in the productivity–precipitation relationship (Estiarte *et al*. 2016). Nonetheless, the rewetting event triggered an increase in fine root biomass after the end of the natural drought. This post‐drought enhancement of root growth (Pregitzer, Hendrick & Fogel 1993) was not altered by DH.

In line with our hypothesis, DH led to alterations in C:N ratios of shoots and fine roots that were mainly due to changes in N content: in shoots, the N concentration was lower, while in fine roots, it was higher than in plants without DH. A similar decrease of N in shoots during drought was reported for other grasslands (Walter *et al*. 2012) and has been suggested to be more pronounced when species diversity is high (Bloor & Bardgett 2012). Nutrient diffusion and substrate supply towards roots may have been restricted in DH plots, as SWC was lower than in AC plots, (Chaves *et al*. 2002; Durand, Gonzalez‐Dugo & Gastal 2010; Manzoni *et al*. 2013) probably due to an incomplete recovery from the preceding experimental drought (Goebel *et al*. 2011). Thus, one might assume that plants in DH plots may have been more strongly limited by N‐supply during the natural drought. However, to our surprise, plants with DH showed higher root N concentrations. This suggests that N‐supply was not limiting root growth and could indicate that N was possibly allocated from shoots to below‐ground organs or stored in roots for increased production of N‐containing osmolytic compounds (Miller 2010). In contrast to other studies, where inorganic soil N pools increased during drought (Bloor & Bardgett 2012; Fuchslueger *et al*. 2014b; Canarini & Dijkstra 2015) inorganic soil N pools decreased in our study. This observation points to the alternative explanation of the increased root N concentration, namely that plants with DH were able to take up more N. Overall, the observed shifts in root vs. shoot biomass and C:N ratios induced by DH indicates increased competition among plants and between plants and microbes for soil N during drought (Ollivier *et al*. 2011; Reyer *et al*. 2013) and could in turn lead to shifts in substrate (plant litter) quality for microbial decomposition (Bardgett, Deyn & Ostle 2009).

In line with our second hypothesis, the observed reduction in ^13^C uptake in shoots in DH plots during the natural drought was even stronger than reductions observed during a previous single, but more severe experimental drought (Hasibeder *et al*. 2015). Compared to plants in AC plots, the allocation speed of fresh C to fine roots was reduced in DH plots, and ^13^C was turned over more slowly during the natural drought indicating strong DH effects. In contrast to other experiments testing effects of single droughts, where the allocation of newly assimilated C to fine roots increased (e.g. Burri *et al*. 2014; Hasibeder *et al*. 2015), DH in our study reduced the proportion of fresh C allocated below‐ground during drought. Moreover, during drought, fine root respiration rates were decreased more strongly in DH than in AC plots, and although ^13^C tracer was detectable in the bulk root biomass, root respiration in DH plots was almost exclusively fuelled by carbon stored before labelling (at least older than 144 h). This suggests that the short natural drought in DH plots had an even stronger impact on the turnover of newly assimilated C in roots than an earlier extreme experimental drought (Hasibeder *et al*. 2015). Hence, newly assimilated carbon may have been used for biomass production or could have been allocated to osmotic compounds to increase water flow towards fine roots to enhance water uptake (Chaves *et al*. 2002; Hasibeder *et al*. 2015). Although the ^13^C recovered in the extractable soil organic C pool was significantly lower in DH plots, still a large amount of fresh C was detectable, which could have been exuded or leaked passively from the roots into the rhizosphere (Walker *et al*. 2003; Jones, Hodge & Kuzyakov 2004; Jones, Nguyen & Finlay 2009). Thus, DH may enhance the effects of drought on ecosystem C cycling by reducing the short‐term coupling of above and below‐ground C turnover.

### Drought History Affects the Functional Response of Microbes to Drought and Rewetting

In our study, microbial biomass was similar in DH and AC plots before the natural drought and increased slightly during drought and subsequent rewetting in DH but not in AC plots. In soils, only a small portion of the total microbial biomass is maintaining an active state at any given time (Blagodatskaya & Kuzyakov 2013). Thus, possible changes in response to drought and rewetting might have been buffered by the comparably large proportion of living, but inactive (or potentially active) microbes. We found, however, that structural changes within the microbial community composition were triggered by both DH and by the natural drought and subsequent rewetting.

First, and in contrast to our hypothesis, we could detect differences in the microbial community composition between DH compared to AC plots before the natural drought; these differences were mainly due to a lower number of fungal biomarkers. This indicates that fungi might be less resilient and may need more time to recover than bacteria, although they were also reported to be more resistant to drought in other studies (Yuste *et al*. 2011; de Vries & Shade 2013).

Secondly, the ratio of fungi:bacteria decreased during the natural drought due to a strong increase of gram‐positive bacterial markers by more than 100%. This suggests that gram‐positive bacteria might have become activated (Blagodatskaya & Kuzyakov 2013) and is further evidence that these bacterial groups may be better adapted to low water potentials during drought (Lennon *et al*. 2012; Fuchslueger *et al*. 2014a; Orwin *et al*. 2015). The incorporation of fresh plant C during the first 48 h after the labelling pulse (during the natural drought) into microbial PLFAs, particularly into bacterial markers was, however, strongly reduced in DH plots. This indicates that the natural drought has affected the activity of microbes more strongly in DH than in AC plots. The reduction may have been primarily caused by the fact that during the natural drought soil moisture was significantly lower in DH as compared to AC plots and may have been further contributed to a comparatively stronger reduction of below‐ground carbon allocation in DH relative to AC plots. Low soil moisture is known to reduce substrate diffusion towards microbes and therefore the availability of fresh C (Manzoni, Schimel & Porporato 2012; Schimel & Schaeffer 2012; Moyano, Manzoni & Chenu 2013; Manzoni *et al*. 2014). Alternatively, different functional groups of microbes were active in DH compared to AC plots, that were less depended on plant‐derived C, as,for example, several groups of gram‐positive bacteria.

Thirdly, within 2 days after the rewetting was induced by a rain event, a further microbial community shift was detectable, which was independent of DH. This fast shift was not related to any particular group (fungal, gram positive or gram negative) of biomarkers. More detailed studies based on rRNA data have, however, shown a sequential resuscitation of different microbial taxa within hours after rewetting (Placella, Brodie & Firestone 2012). The much coarser resolution of PLFAs in our study may thus not have been able to cover the fast microbial such dynamic during rewetting (Blagodatskaya & Kuzyakov 2013). Notably, the rewetting led to a strong increase of ^13^C in all microbial groups only in DH plots, indicating that similar as shown by others microbes regained activity (Or *et al*. 2007; Borken & Matzner 2009; Placella, Brodie & Firestone 2012; Barnard, Osborne & Firestone 2013; Meisner, Rousk & Bååth 2015) and used accumulated plant‐derived carbon to produce PLFAs as has been previously described by Fuchslueger *et al*. (2014a). In contrast, in AC plots, ^13^C seems to have been incorporated more rapidly during the drought period. This and the generally faster turnover in PLFA biomarkers in AC plots indicates that no critical threshold exists for the interaction between plant and the microbial community above the permanent wilting point.

Overall, our data suggest that DH neither improved nor hampered the structural responses of microbes to drought and rewetting and that microbial responses to recurrent drought and rewetting are highly dynamic (Cruz‐Martínez *et al*. 2009; Landesman & Dighton 2010; Blazewicz, Schwartz & Firestone 2014). Nevertheless, our results also indicate that DH changes microbial turnover of plant‐derived C during subsequent drought events.

## Conclusions

Our study showed that although a history of extreme drought periods did not affect the response of grassland productivity to a subsequent drought, DH clearly changed the drought responses of shoot and root C:N ratios, C assimilation and below‐ground C allocation, as well as fine root C metabolism. Hence, the projected increases in the frequency of drought periods could, in the long term, alter the quality of plant C inputs for microbial decomposition and thus affect ecosystem C turnover and storage.

Moreover, we conclude that DH did not affect the response of the microbial community structure to reduced soil moisture, as reflected by a similar increase of gram‐positive bacteria independent of DH. In contrast, the functional response of microbes to low soil moisture, that is the incorporation and turnover of recent plant‐derived C, was strongly decreased by DH. We therefore conclude that the legacy of DH strongly affects microbial responses to further drought events with repercussions on ecosystem carbon dynamics in a changing climate.

## Data accessibility

Data deposited in the Dryad repository: http://dx.doi.org/10.5061/dryad.2t3sn (Fuchslueger *et al*. 2016).

## Supporting information


**Appendix S1.** Description of the different models used for determination of ^13^C turnover in plants, soil and microbial PLFAs.
**Table S1.** Microclimatic conditions during ^13^C pulse‐labelling.
**Table S2.** Nonlinear regressions describing the turnover of ^13^C through the plant‐soil system that were used for the determination of mean residence times of ^13^C in sampled compartments.
**Fig. S1.** Soil parameters in AC (black) and DH plots (grey) before (pre‐dry), during, during the recurring dry period (dry) and after a subsequent heavy rain pulse (wet).
