# Supplementary material for: Drought history affects grassland plant and microbial carbon turnover during and after a subsequent drought event
Source: J Ecol. 2016 May 24;104(5):1453–65. doi: 10.1111/1365-2745.12593 (PMC4996329; doi:10.1111/1365-2745.12593)
Supplement: Supplementary file 1 — Appendix S1. Description of the different models used for determination of 13C turnover in plants, soil and microbial PLFAs. Table S1. Microclimatic conditions during 13C pulse‐labelling. Table S2. Nonlinear regressions describing the turnover of 13C through the plant‐soil system that were used for the determination of mean residence times of 13C in sampled compartments. Fig. S1. Soil parameters in AC (black) and DH plots (grey) before (pre‐dry), during, during the recurring dry period (dry) and after a subsequent heavy rain pulse (wet). [file JEC-104-1453-s001.docx]

**Supporting information:**

***Description of the different models used for determination of ^13^C turnover in plants, soil and microbial PLFAs:***

Plant shoots are the primary compartment to take up ^13^C, thus after a labelling pulse they follow a typical exponential decay curve dominated by net ^13^C export (Epron et al., 2012; Studer et al., 2014), as ^13^C can be transferred to other plant compartments or is lost through respiration:

$N\left( t \right)=N_{0}*e^{(-k*t)}$ *(Exponential decay)* **(1)**

with *N_0_* is the amount of label at the ^13^C peak 1.5 hours after labelling, *k* is decay constant and *t* is time after the labelling pulse.

The other compartments (plant roots, root respiration, EOC, and microbial marker) follow a different pattern, which is more similar to label dynamics during continuous labelling (Studer *et al.* 2014). After the labelling pulse the ^13^C accumulates after a certain lag-phase either exponentially, or follows a sigmoidal curve to a peak or a plateau and then decreases either exponentially or linearly (Epron *et al.* 2012; Studer *et al.* 2014):

$N\left( t \right)=N_{0}+a*e^{\left( -k_{1}*t \right)}+k_{2}*t$ *(Exponential increase with linear decrease)* **(2)**

with *N_0_* is the ^13^C pool at time 0, *a* is the peak amount of ^13^C, *k_1_* is an accumulation constant during the accumulation phase, *k_2_* is the decay constant after the ^13^C label peak, and *t* is the time after the labelling pulse. To determine the mean residence time we only considered the decay constant *k_2_*. As in drought-history plots the microbial PLFA markers were still in the accumulation period we alternatively used an exponential increase curve to determine the mean accumulation time:

$N\left( t \right)=N_{0}*e^{(k*t)}$ *(Exponential increase)* **(3)**

with *N_0_* describing the initial amount of ^13^C, *k* is the accumulation constant, *t* is the time after labelling. As the peak was not yet reached no mean residence time was calculated.

**References:**

Epron, D., Bahn, M., Derrien, D., Lattanzi, F., Pumpanen, J., Gessler, A., Hogberg, P., Maillard, P., Dannoura, M., Gerant, D. & Buchmann, N. (2012) Pulse-labelling trees to study carbon allocation dynamics: a review of methods, current knowledge and future prospects. *Tree Physiology*, **32**, 776–798.

Studer, M., Siegwolf, R. & Abiven, S. (2014) Carbon transfer, partitioning and residence time in the plant-soil system: a comparison of two 13CO2 labelling techniques. *Biogeosciences*, **11**, 1637–1648.

**Table S1:** Microclimatic conditions during ^13^C pulse-labelling. *Date* and time of the day (*Time*) of ^13^CO_2_ pulse-labelling for pairs of *control* and *post-drought* plots, respectively. *^13^CO_2_* describes the average isotopic composition in the labelling chamber given in atom% ^13^C during labelling analysed by a Quantum Cascade Laser (QCL, Aerodyne Research Inc., Billerica, MA, USA); the average amount of *^13^CO_2_ added* is given in ml min^-1^. Photosynthetic active radiation (PAR in *µmol m^-2^s^-1^*) during labelling as well as air temperature inside (*T_in_*) and outside (*T_ou_*_t_) the chambers are displayed (minimum – maximum temperature, in °C).

| **Date** | **t** | **Time** | **^13^CO_2_** | **^13^CO_2 add_** | **PAR** | **T_out_** | **T_in_** |
| --- | --- | --- | --- | --- | --- | --- | --- |
| 23.08.2011 | *DH* | 11:25 | 23.5 ± 1.7 | 30.3 | 1539–1820 | 18.8–24.2 | 22.8–33.2 |
|  | *AC* | 11:55 | 22.9 ± 1.9 | 43.2 | 1539–1820 | 20.5–24.4 | 24.9–30.1 |
| 24.08.2011 | *DH* | 10:32 | 18.4 ± 2.7 | 13.5 | 1500–1730 | 26.0–30.9 | 26.7–37.7 |
|  | *AC* | 11:09 | 23.2 ± 1.3 | 37.9 | 1500–1730 | 26.0–28.8 | 28.8–39.9 |
| 25.08.2011 | *DH* | 10:24 | 22.6 ± 1.4 | 23.1 | 1320–1670 | 15.7–24.1 | 15.7–24.1 |
|  | *AC* | 10:54 | 19.0 ± 1.9 | 21.4 | 1320–1730 | 14.9–25.8 | 14.9–25.8 |

**Table S2:** Nonlinear regressions describing the turnover of ^13^C through the plant-soil system that were used for the determination of mean residence times of ^13^C in sampled compartments. Asterisks mark levels of significance (°p<0.0.1; *p<0.05; **p<0.01; ***p<0.001).

|  | **AC** | | | | **DH** | | | |
| --- | --- | --- | --- | --- | --- | --- | --- | --- |
|  | function | R^2^ | F | p | function | R^2^ | F | P |
| Shoots | N(t)=381.7*exp^(-0.014*t)^ | 0.44 | 15.38 | ******* | N(t)=170.9*exp^(-0.015-t)^ | 0.30 | 9.69 | ** |
| Fine roots | N(t)=38.4-31.7*exp^(-0.115*t)^-0.1017*t | 0.34 | 3.42 | ***** | N(t)= 8.3-8.3*exp^(-1.21*t)^-0.0087*t | 0.16 | 1.4 | ns |
| Fine Root Resp. | N(t)= 0.4 -0.4*exp^(-1.175*t)^-0.0023*t | 0.36 | 3.95 | ***** | na |  |  |  |
| EOC | N(t)= 2.9 -3.0*exp^(-0.204*t)^-0.0081*t | 0.45 | 5.35 | ****** | N(t)= 2.0-2.2*exp^(-0.314*t)^-0.0072*t | 0.20 | 1.79 | ns |
| PLFA_tot_ | N(t)= 6.4 -6.6*exp^(-0.209*t)^-0.041*t | 0.29 | 2.82 | ns | N(t)= 0.38*exp^(0.024*t)^ | 0.69 | 48.52 | *** |
| Fungi_tot_ | N(t)= 2.3 -2.4*exp^(-0.155*t)^-0.015*t | 0.41 | 4.59 | * | N(t)= 0.15*exp^(0.021*t)^ | 0.66 | 42.22 | *** |
| Bacteria_tot_ | N(t)= 1.5 -1.4*exp^(-0.270*t)^-0.009*t | 0.19 | 1.71 | ns | N(t)= 0.01*exp^(0.043*t)^ | 0.66 | 43.03 | *** |
| Gram-positive | N(t)= 0.5 -0.5*exp^(-0.228*t)^-0.003*t | 0.22 | 1.87 | ns | N(t)= 0.002*exp^(0.048*t)^ | 0.63 | 38.03 | *** |
| Gram-negative | N(t)= 1.3 -1.2*exp^(-0.144*t)^-0.009*t | 0.29 | 2.70 | ns | N(t)= 0.007*exp^(0.041*t)^ | 0.67 | 45.52 | *** |


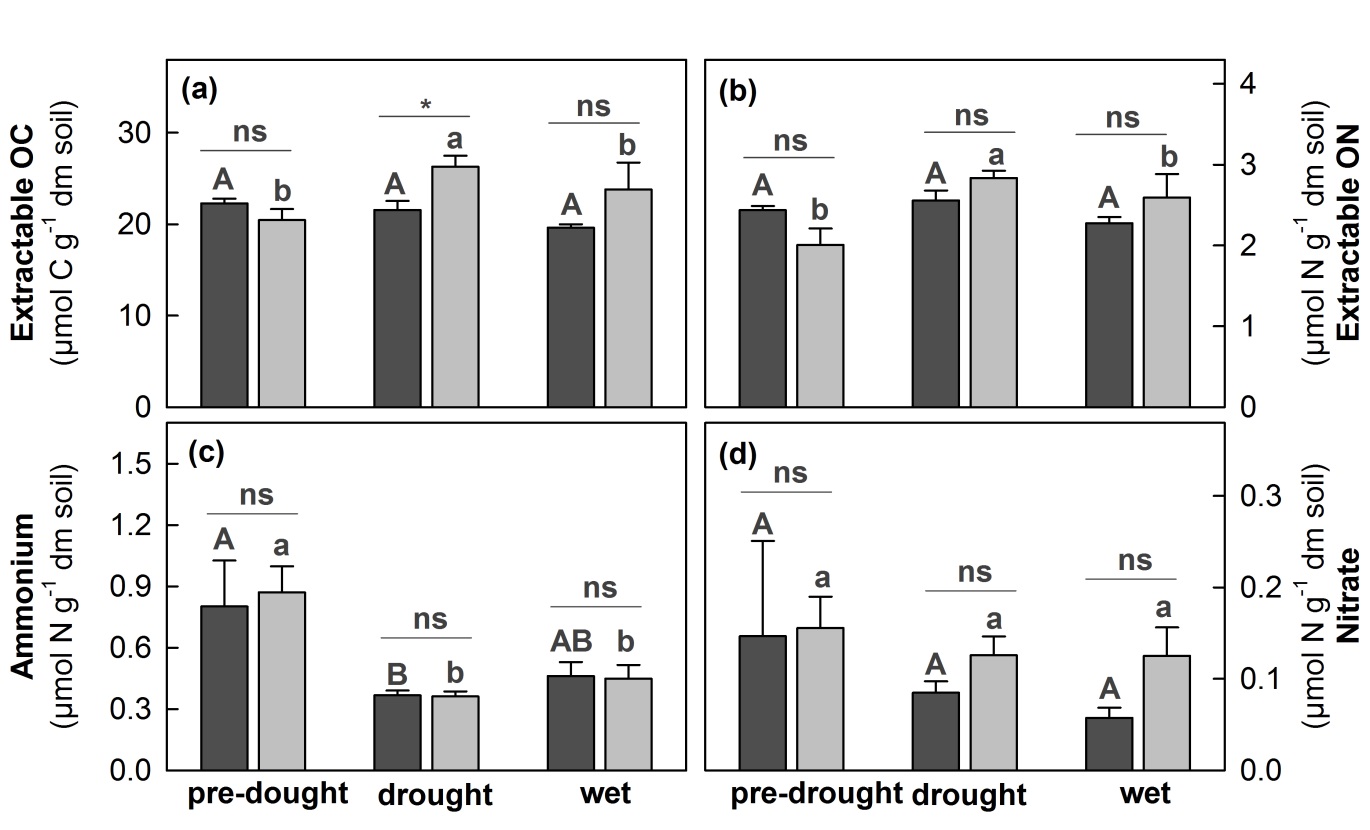


**Figure S1:** Soil parameters in AC (black) and DH plots (grey) before (pre-dry), during, during the recurring dry period (dry) and after a subsequent heavy rain pulse (wet). Means of (a) soil extractable organic carbon and (b) extractable organic nitrogen, (c) ammonium and (d) nitrate concentrations were determined (n=3). Significant differences between AC and DH at single samplings are marked with asterisks (*p<0.05; **p<0.01); differences at a 0.05 significance level between pre-dry, dry and wet conditions within AC are marked with upper case letters, while differences within DH treatment are marked with lower case letters; see Table 1 for details.
